# Predicting the survival of kidney transplantation: design and evaluation of a smartphone-based application

**DOI:** 10.1186/s12882-022-02841-4

**Published:** 2022-06-21

**Authors:** Leila Shahmoradi, Alireza Borhani, Mostafa Langarizadeh, Gholamreza Pourmand, Ziba Aghsaei fard, Sorayya Rezayi

**Affiliations:** 1 Department of Health Information Management and Medical Informatics, School of Allied Medical Sciences, Tehran University of Medical Sciences, Tehran, Iran; 2 Department of Health Information Management and Medical Informatics, School of Health Management and Information Science, Iran University of Medical Sciences, Tehran, Iran; 3 Urology Research Center, Tehran University of Medical Sciences, Tehran, Iran

**Keywords:** Chronic kidney disease, Kidney transplant, Survival, Usability evaluation

## Abstract

**Background:**

Prediction of graft survival for Kidney Transplantation (KT) is considered a risky task due to the scarcity of donating organs and the use of health care resources. The present study aimed to design and evaluate a smartphone-based application to predict the survival of KT in patients with End-Stage Renal Disease (ESRD).

**Method:**

Based on the initial review, a researcher-made questionnaire was developed to assess the information needs of the application through urologists and nephrologists. By using information obtained from the questionnaire, a checklist was prepared, and the information of 513 patients with kidney failure was collected from their records at Sina Urological Research Center. Then, three data mining algorithms were applied to them. The smartphone-based application for the prediction of kidney transplant survival was designed, and a standard usability assessment questionnaire was used to evaluate the designed application.

**Results:**

Three information elements related to the required data in different sections of demographic information, sixteen information elements related to patient clinical information, and four critical capabilities were determined for the design of the smartphone-based application. C5.0 algorithm with the highest accuracy (87.21%) was modeled as the application inference engine. The application was developed based on the PhoneGap framework. According to the participants’ scores (urologists and nephrologists) regarding the usability evaluation of the application, it can be concluded that both groups participating in the study could use the program, and they rated the application at a "good" level.

**Conclusion:**

Since the overall performance or usability of the smartphone-based app was evaluated at a reasonable level, it can be used with certainty to predict kidney transplant survival.

**Supplementary Information:**

The online version contains supplementary material available at 10.1186/s12882-022-02841-4.

## Background

Chronic Kidney Disease (CKD) is a gradual loss of kidney function that can occur due to diabetes, high blood pressure, recurrent infections, and urinary tract obstruction [1]. Unlike acute renal failure, which progresses rapidly and is potentially reversible, CKD is a long-term disease in which kidney damage is permanent and progressive [2]. Based on the 2010 Global Burden of Disease (GBD) investigation, CKD was ranked 27^th^ in the list of the causes of deaths worldwide in 1990, but it was ascended to rank 18^th^ in 2010 report [3]. This disease can progress to ESRD, which is fatal without dialysis or KT [4].

Nowadays, KT is the most effective treatment for advanced and irreversible renal failure [5]. According to the investigation done on the WHO data (2008) in 104 countries accounting for 90% of all transplantations across the world, about 100,800 organ transplants have been performed in these countries, and there have been 69,400 cases of KT (over 68% of all transplants in the world, from which 46% have been carried out with the live donors), [6]. This procedure is remarkably cost-effective and imposes fewer complications on patients compared to dialysis [7]. Kidney graft rejection has many consequences, such as re-transplantation or even death [8]. The problem of graft rejection is mainly attributed to chronic allograft rejection associated with dysfunctional donor-recipient matching [9]. Based on the literature, kidney graft survival prediction is essential for the transplant success, since it both increases the effective use of healthcare system's resources and the utility of available organs [9–11].

The increasing use of new data mining techniques, especially to discover unique patterns, has become widespread in the medical industry [12, 13]. These methods could be applied as adjunct tools to predict survival of graft transplantation [14]. Along with the use of data-based algorithms, Information Communication Technology (ICT) has revolutionized the e-health industry. Among the various ICT tools, wireless and smartphone-based technologies offer opportunities to reduce costs and raise access to health services, which improve the effectiveness of healthcare delivery process [15]. Thus, the integration of wireless technologies, data mining and machine learning approaches leads to effective delivery of care and provision of unique diagnostic services to individuals [16].

Previous studies have established several effective prediction data-driven models to identify kidney post-transplantation graft survival rates [9, 10, 17]. Our previous study dealt with data and modeling to predict post-transplantation graft survival among kidney transplant recipients using data mining algorithms [18]. In the present study, C5.0 algorithm with the highest accuracy (96.77%) was the chosen model for predicting patients' kidney transplant survival. In this way, the output of these results can be monitored through a smartphone or a tablet application that is easily accessible. Appropriate medical decisions can also be made based on these results. Thus, the main purpose of this study was to design and evaluate a smartphone-based application to predict the survival of KT for specialists and patients. To evaluate the application's usability, we used a standard questionnaire. In the implementation phase, the beneficiaries of this application included urologists, nephrologists, and kidney transplant patients. The primary use of the smartphone-based application is to help professionals predict the survival of KT. Also, there are facilities in this software through which, patients can use the existing reminders for appointments and set the new version of the reminders for suppressive drugs and post-transplant care instructions.

### Implementation

This is an applied-developmental study that was conducted by Tehran University of Medical Sciences. This study was conducted in three main phases:

#### First phase of the study

The initial parameters in predicting post-transplantation graft survival among kidney transplant recipients were determined by reviewing specialized textbooks and consulting with supervisors and a clinical consultant. Based on the initial review, a researcher-made questionnaire was developed to assess the information needs of the application through urologists and nephrologists. This questionnaire was distributed among specialists (three urologists and four nephrologists) working at Sina Hospital. Participants were asked about data items and the capabilities required by an application to predict kidney transplant survival. The convenience sampling method was used to select the research sample. Each of the required data items was considered essential if an average of 50% of the respondents recognized it as necessary, which was then used in the application design. The reliability of the questionnaire was measured by test–retest method so that, after a short time, the questionnaire was given to the same people to be completed by them again. The scores obtained from the two tests were examined and the correlation coefficient of 92% was obtained. The questionnaire helped to prepare a checklist by which, further extraction of information was performed. Using this checklist, the medical records of 513 kidney transplant patients at Sina Urological Research Center were reviewed and main features were identified.

#### Second phase of the study

After collecting the input data items related to each patient by checklists, and also modifying the data to reduce the modeling error, useful features were extracted to predict the survival of KT. The predictive models such as C5.0, C&R, and neural network algorithms were used. The details and results of training and testing processes have been provided in our previous study [19]. In order to design and implement a multi-level smartphone application for predicting the survival of KT, the PhoneGap framework was used, which was responsible for communicating with the hardware and smartphone operating system. After installing the PhoneGap framework, Android and iOS platforms were run on it. The coding of programs’ display was done by Hypertext Markup Language (HTML) and Cascading Style Sheets (CSS), which included the formatting and components of the application’s pages. Also, JavaScript and its more advanced codes, such as jQuery, were employed.

#### Third phase of the study

**Fig. 1:**
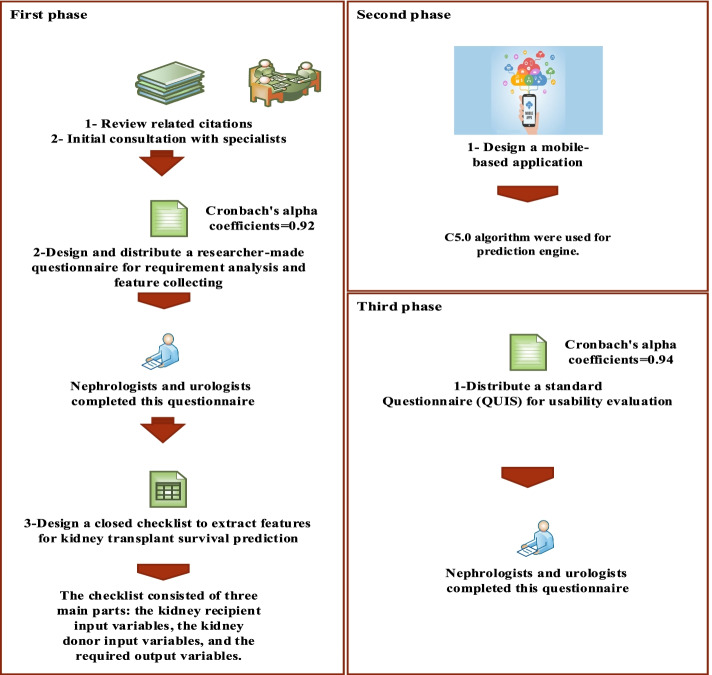
Phases of the study at a glance (the figure is illustrated by the authors)

In the last phase, a standard questionnaire was used to evaluate the usability of the final version of the application [20]. The chosen standard questionnaire was the Questionnaire for User Interface Satisfaction (QUIS), which elicits users’ opinions and evaluates users’ acceptance of the application interface. Validity of the questionnaire was confirmed by face validity and also taking into account the opinions of five specialists, thus its reliability was reported at ɑ = 0.94. Figure 1 shows different phases of the study separately.

## Results

### Information elements, main features and models’ performance

The frequency distribution of responses from urologists and nephrologists regarding the required data elements (in the researcher-made questionnaire) are presented in supplement Table S1. Based on the results of the questionnaire, a checklist was created by which data were extracted from the records of patients (513 cases). We reached the compelling features that were utilized to predict the survival of KT, which are presented in Table 1.

**Table 1:** Features used for predicting kidney transplant survival in applied algorithms

#	Features	Note	Type
1	ESRD	Cause of kidney failure	Nominal
2	R-AGE	Age of the kidney recipient	Numerical
3	BMI	Body mass index of the recipient	Numerical
4	R-SEX	Sex of the kidney recipient	Nominal
5	TYPE-DIALYSIS	Duration of dialysis before transplantation	Numerical
6	PANEL-TEST	Panel Test	Numerical
7	FIRST-TRANSPLANT	History of transplantation	Nominal
8	RELATIONSHIP	Type of communication between recipient and donor of the kidney (relative, non-relative, corpse)	Nominal
9	D-AGE	Age of the kidney donor	Numerical
10	D-SEX	Gender of the kidney donor	Nominal
11	SURVIVAL-YEAR	The duration of transplant survival in years	Nominal

The three predictive data mining models of C5.0 Decision Tree, neural network, and C&R Tree were analyzed and modeled, using IBM SPSS Modeler 14.2. Data of the database were randomly divided into two parts; 70% (360 cases) for training and 30% (153 cases) for testing. Details of the training/testing results of the algorithms have been given in the previous study [18].

In summary, the accuracy of C&R Tree and neural network models for the training dataset were estimated at 83.7% and 79.5%, respectively. Also, the accuracy of survival rate reached to 96.77% by C5.0. Additionally, the performance metrics of the predictive models for testing dataset, sensitivity, specificity, and accuracy are provided in the supplement Table S2. The highest accuracy rate belonged to the C5.0 model (87.21%).

### Designing the application

The PhoneGap framework was used to design a multi-level smartphone application for predicting kidney transplant survival. In this section, the rules of C5.0 model algorithm, which has the highest accuracy among other models in evaluating data mining models, were used for coding. This application has various sections. Some of these sections are presented below:

The main pages of the application are depicted in supplement Figs. 1 and 2.

**Fig. 2:**
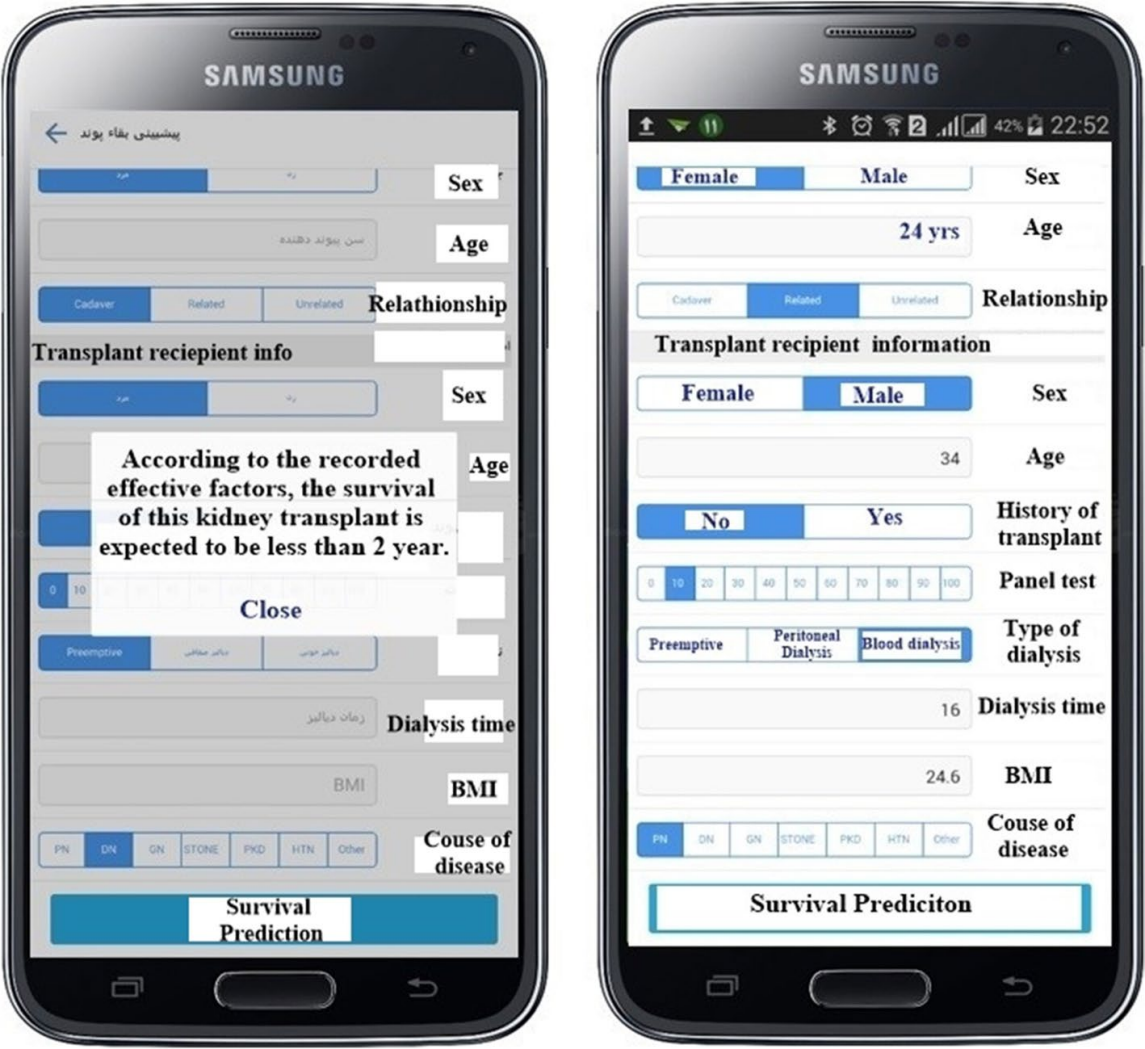
Prediction of kidney transplant survival using C5.0 algorithm (the figure is illustrated by the authors)

The third button shows the main goal of the research, which is used to predict kidney transplant survival, and its target users are the specialists (Fig. 2). The prediction result is numerically shown to the specialist, which is between 1 and 7 years.

### Usability evaluation of the application

**Fig. 3:**
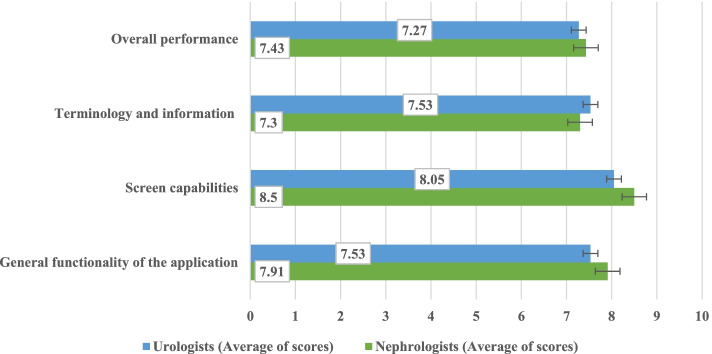
Average responses of the participants in regard to usability evaluation of the application

We used QUIS for evaluating the usability of the application. Figure 3 shows the average responses of the participants (urologists and nephrologists) regarding the usability evaluation of designed application.

As seen in the figure above, considering the mean scores obtained for the overall performance, terminology and information used in the app, screen capabilities, and general functionality, which are in the category of 6–9, it can be concluded that both groups participating in the study were able to use the program, and they rated the application at a "good" level.

## Discussion

To create an application for predicting kidney transplant survival, information needs and vital capabilities of the application were extracted. Regarding patients' personal information, only three items of full name, address, and telephone number were included in the checklists to contact patients and inform them about the survival rate of KT if needed. On the other hand, results of the questionnaire completed by the nephrologists and urologists showed that in terms of the patient clinical information, the most important parameters influencing the survival of kidney transplant included; cause of kidney failure, body mass index, type of dialysis, age of the kidney recipient, height of the kidney recipient, weight of the kidney recipient, gender of the kidney recipient, duration of preoperative dialysis, panel test, history of transplantation, previous diseases such as kidney stones, the relationship between donor and recipient, and gender and age of the kidney donor.

In a study to predict the survival of KT, patients' blood samples were used and 18 laboratory variables were analyzed among 108 patients with end-stage renal disease [21]. Also, to predict chronic allograft kidney disease, 23 laboratory variables in the blood and urine of 80 transplant patients were analyzed six months after the transplantation [22]. However, clinical-laboratory items were deleted in our study and were not used in our study after consulting with a clinical consultant.

In the present study, after creating an application to predict the survival of KT, the usability test and assessment of user satisfaction were performed. The usability evaluation in this study was carried out by the standard Chin test [23]. In line with our study, in some studies, the users were asked to indicate the capabilities needed in the smartphone application to predict transplant survival, and their ideas were applied in the design of the application [2, 24–26].

In a study entitled: “The decision tree in the follow-up of kidney transplantation”, the survival or rejection of a kidney transplantation was predicted using binary tree at four levels, and also the sensitivity and data of the Greco tree questionnaire were estimated to be 88.2% and 73.8%, respectively [11]. However, in the present study, a graft survival prediction model was developed using division and regression tree or C&R, and a weight was given to each of the input factors. Thus, with the production of 20 C&R trees and the combination of connections between these models, the optimal model with an accuracy of 83.7% was estimated.

In our research, to evaluate the application, seven people were surveyed and the mean scores obtained by them indicated that they evaluated the program at a "good" level. In general, it can be said that there are standard questionnaires and different methods for assessing the usability of applications [27], which are used depending on the type of research and the opinion of researcher [25]. A mHealth program was designed to help the early detection of CKD and self-monitoring according to quality characteristics, such as safety, efficacy, and usability. KaPA value of 0.7119 showed a high agreement between the program and the three neurologists, and also the level of usability of the program was evaluated at a good level [26].

One of the strengths of this study is the surveying of specialists in the first phase of the study to identify the information elements/features and capabilities of the application. This can increase the generalization of our work. This app can help urologists and nephrologists, because its main use is to help professionals predict transplant survival. On the other hand, there are other features in this application through which patients can utilize the reminders for clinical appointments and also receive a newer version of the reminder, suppressive drugs prescription, and post-transplant care instructions.

One of the limitations of this study is that, the application was designed based on data obtained from only one hospital. It is suggested to use the data of other research centers in future studies and compare the results. On the other hand, the geographical area in which the research institute is located is significantly important, because factors such as level of well-being, social class, and employment of the donor and recipient of the kidney can affect the survival of KT. However, such factors were not considered in this study, as such information was not available. In this study, one of the most important limitations is that due to the time and budget constrain, we could not evaluate the patients’ perspective in the usability evaluation phase, and only entered the specialists’ perspective in the evaluation phase. The usability evaluation of the designed application by patients is suggested in future studies.

## Conclusion

In this study, information items and software capabilities were identified, and the application was designed to predict post-transplantation graft survival among kidney transplant recipients. The main parts of the application included post-transplant care, reminder, survival prediction, about us, setting, and log out. The button of “survival prediction” is used to predict kidney transplant survival, and its target users are the specialists. Based on the average scores obtained for the overall performance, terminology and information used in the app, screen capabilities, and general functionality, the app was rated by the participants at a "good" level.

## Supplementary Information

**Additional file 1. **

## Data Availability

The dataset used and analyzed during the current study are available from the corresponding author on reasonable request.

## Abbreviations

ESRD: End-Stage Renal Disease
CKD: Chronic Kidney Disease
KT: Kidney Transplantation
HTML: Hypertext Markup Language
CSS: Cascading Style Sheets
ICT: Information Communication Technology
QUIS: Questionnaire for User Interface Satisfaction
C&R: Classification and Regression
ENT: Ears, Nose and Throat
K.U.B: Kidney, Ureter, and Bladder
VCUG: Voiding Cystourethrogram
BMI: Body Mass Index
GI: Glycemic Index
