# Supplementary material for: Predicting the survival of kidney transplantation: design and evaluation of a smartphone-based application
Source: BMC Nephrol. 2022 Jun 21;23:219. doi: 10.1186/s12882-022-02841-4 (PMC9210621; doi:10.1186/s12882-022-02841-4)
Supplement: Supplementary file 1 — Additional file 1. [file 12882_2022_2841_MOESM1_ESM.docx]

**Supplement material**

Frequency distribution of responses are presented in Table S1. The required data items were considered necessary if on average 50% of the respondents considered it necessary (blue items were selected). The performance metrics of the predictive models for testing dataset, sensitivity, specificity, and accuracy is provided in Table S2.

**Table S1.** Frequency distribution of responses

| The number of participants who identified the item as necessary | Necessity average (%) | Questionnaire items |
| --- | --- | --- |
| - | - | 1. **Patient demographic information** |
| 7 | 100 | 1-1. Fist name/Last name |
| 3 | 43 | 1-2. National code |
| 2 | 28 | 1-3. Address |
| 7 | 100 | 1-4. Phone number |
| 3 | 43 | 1-5. Economic situation |
| 5 | 72 | 1-6. Record number |
| - | - | 1. **Patient clinical information** |
| 7 | 100 | 2-1. Gender of kidney recipient |
| 7 | 100 | 2-2. Gender of kidney donor |
| 7 | 100 | 2-3. Height and weight of the transplant patient |
| 7 | 100 | 2-4. Age of kidney recipient |
| 7 | 100 | 2-5. Age of kidney donor |
| 3 | 43 | 2-6. Blood type |
| 2 | 28 | 2-7. Duration of hospitalization |
| 6 | 86 | 2-8. Type of dialysis |
| 6 | 86 | 2-9. Duration of dialysis |
| 5 | 72 | 2-10. PANEL-TEST |
| 4 | 57 | 2-11. Year of transplantation |
| 6 | 86 | 2-12. History of transplantation |
| 3 | 43 | 2-13. Kidney weight |
| 7 | 100 | 2-14. Cause of ESRD |
| 3 | 43 | 2-15. Diseases such as cancer and so on |
| 7 | 100 | 2-16. Transplant from donor (alive or brain death) |
| 5 | 72 | 2-17. Cause of donor death |
| 2 | 28 | 2-18. Nephrectomy (left or right) |
| 7 | 100 | 2-19. Relationship with the donor (Related or Unrelated) |
| 2 | 28 | 2-20. Results of dental consultation |
| 3 | 43 | 2-21. Family history of renal failure |
| 1 | 14 | 2-22. Ears, Nose and Throat (ENT) consulting Results |
| 2 | 28 | 2-23. History and physical examinations |
| 1 | 14 | 2-24. Abdominal ultrasonography |
| 2 | 28 | 2-25. Kidney, Ureter, and Bladder (K.U.B) X-ray |
| 1 | 14 | 2-26. A voiding cystourethrogram (VCUG) |
| 1 | 14 | 2-27. Mammography |
| 3 | 43 | 2-28. Cardiac counseling results |
| 2 | 28 | 2-29. Glycemic Index (GI) |
| 7 | 100 | 2-30. Transplant patient status (Alive or dead) |
| 7 | 100 | 2-31. Survival time of transplanted kidney |
| - | - | 1. **Application capabilities** |
| 3 | 43 | 3-1. Ability to display time and date |
| 7 | 100 | 3-2. Show survival rate by year |
| 5 | 72 | 3-3. Use a warning to renew a prescription |
| 5 | 72 | 3-4. Use reminders to take the patient medication |
| 6 | 100 | 3.5. A guide to post-operative kidney transplant training |

**Table S2.** Performance metrics of predictive models

| Performance of Predictive models (for test dataset) | Accuracy | Sensitivity | Specificity |
| --- | --- | --- | --- |
| C5.0 Decision Tree | 87.21% | 90.85% | 52% |
| C&R Tree | 83.28% | 86.85% | 57.28% |
| Neural network | 83.71% | 87.14% | 65% |

**Main Page of the application**

The main page contains six buttons that are visible in the middle of the page (Supplement Figure 1 (a)). Also, Supplement Figure 1 (b) shows some shortcuts for access, settings, and about us options.

**Figure 1 (b) Figure 1 (a)**


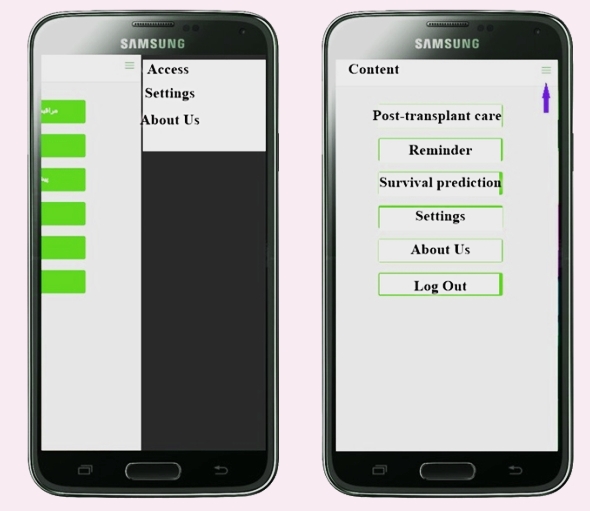


**Supplement Figure 1: (a):** Application homepage **and (b):** Shortcuts to settings and about us

**Post-transplant care**

The first button in the application's main menu is called; "post-transplant care." Clicking on this button will take us to a page with 14 titles, which each one provides brief instructions on post-transplant care to the transplant patient. Supplement Figure 2 (a) shows the main topics of post-transplant care, and Supplement Figure 2 (b) shows the training contents of the application after clicking on the topic, "transplantation and rejection." As it can be seen, in the last section of these topics, the definitions and terms that you will come across in post-transplant care are listed.

**Figure 2 (b) Figure 2 (a)**


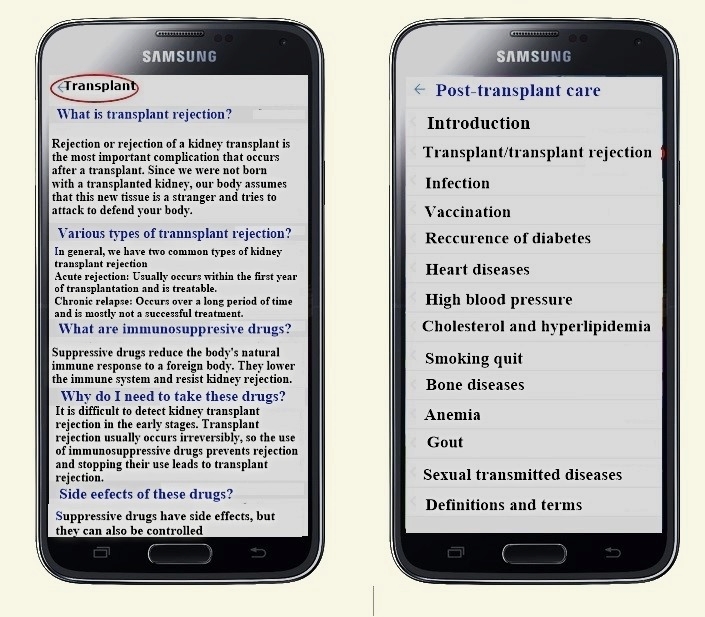


**Supplement Figure 2: (a):** Main topics of post-transplant care**, and (b):** Educational contents of the application after clicking on the topic; "transplantation and rejection”.
